# Instruments used to assess quality of life of older adults in African countries: a scoping review

**DOI:** 10.1186/s12877-021-02262-2

**Published:** 2021-06-05

**Authors:** Priscilla Y. A. Attafuah, Irma H. J. Everink, Ruud J. G. Halfens, Christa Lohrmann, Aaron Abuosi, Jos M. G. A. Schols

**Affiliations:** 1 School of Nursing and Midwifery, University of Ghana, Legon, Ghana; 2 Department of Health Services Research and Care and Public Health Research Institute (CAPHRI), Maastricht University, Maastricht, the Netherlands; 3 Institute of Nursing Science, Medical University of Graz, Graz, Austria; 4 Department of Family Medicine, Care and Public Health Research Institute (CAPHRI), Maastricht University, Maastricht, The Netherlands

**Keywords:** Instruments, Quality of life, Illiterate older people, Scoping review, Slum

## Abstract

**Background:**

Over 60% of the population in sub-Saharan Africa, live in informal settlements (slums) with little or no resources. To be prepared to meet the needs of older people living in slums, it is necessary to know more about their quality of life (QoL). The objective of this review is to identify instruments, which can be used by researchers to assess the QoL of older adults living in African countries, especially those dwelling in slums.

**Methods:**

A scoping review was performed using the databases Scopus, PubMed, and ISI Web of Science to retrieve studies published from January 2008 – September 2020. Studies were included if they reported generic QoL instruments, focused on adults with a mean age ≥ 50 and were conducted in African countries.

**Results:**

In total, 18 studies were included using 7 unique instruments to measure QoL (EUROHIS-QOL-8, SWLS, WHOQOL-OLD, the WHOQOL-BREF, SF-36, SF-12 and RAND-38). All instruments could be interviewer-administered and had 5–36 items. However, little is known about their psychometric properties (validity and reliability), time-investment and cultural sensitivity of the domains included in the instruments.

**Conclusions:**

Even though this review retrieved instruments used to assess QoL of older adults in African countries, there is a need for further research on adjustment and validation of currently existing QoL instruments. In addition, the development and validation of a new instrument which can be used in (illiterate) older populations, living in slums in Africa should be considered.

## Background

Evidence suggests that over 60% of the population in sub-Saharan Africa lives in informal settlements (slums) with little or no resources [1–5]. While formal settlements are usually equipped with good houses, sanitation and services such as hospitals, market places and schools, slums lack these amenities. Slum dwellers have to deal with poor quality of housing (such as wooden or metal structures or containers), lack of sanitation, overcrowding, extreme environmental hazards such as choked gutters, burning and improper disposal of waste. Also, educational facilities or health care services are absent in slums [6, 7]. This causes a significant threat to both health status and life expectancy of slum dwellers [2, 8, 9]. Also, research showed that older adults living in slums are dealing with anxiety about poor prospects of their children, aggression and disrespect from younger generations [4]. These factors, coupled with high illiteracy rate, poor socioeconomic status and high level of spirituality among the African population, can make older adults more religious as they cling on to faith to survive [53]. It is common in Ghana, to seek spiritual assistance from pastors, imams, traditionalists for most health problems rather than patronizing the hospital.

The population aged 60 years and above in Africa is projected to be 10% of the total population by 2050 [10] and also slums are becoming more populated with older adults. It is likely that the deplorable circumstances slum dwellers have to deal with are especially rough for them as they often deal with physical decline and a need for health and social care. To effectively develop interventions that focus on the needs of older adults, it is imperative to understand their quality of life (QoL).

The WHO defines Quality of Life (QoL) as ‘individuals perception of their position in life in the context of the culture and value systems in which they live and in relation to their goals, expectations, standards and concerns’ [11]. This wide concept encompasses one’s physical health, psychological health, level of independence, social/family relationships, the quality of the living environment and personal beliefs [11].

To gain more insight into the QoL of older people living in slums, validated instruments with good psychometric properties which are culturally appropriate are needed. However, there is little to no research in the area of QoL of the ageing population in Africa, and in slums in particular [12]. Available instruments for assessing QoL are generally developed and validated in high-income countries, which have different socio-cultural, environmental and economic characteristics and different life standards compared to African countries. Additionally, the majority of older adults living in slums are illiterate, making it difficult to use QoL instruments where users have to read or write. Therefore, this study aims to identify instruments which are used in African countries to assess the QoL of older adults, especially those dwelling in slums.

## Methods

### Study design and framework

For this study, a scoping review was performed. We followed the first five steps described by Levac et al. on how to perform scoping reviews [13]. These recommendations are outlined as follows: 1) identifying the research question; 2) identifying relevant studies; 3) study selection; 4) data extraction; 5) analysis, reporting results, and considering the implications of study findings to policy, practice, or research.

### Research question

The research question guiding this scoping review is: Which instruments are available for assessing quality of life among older adults in African countries?

### Identification of relevant studies

The main literature search was conducted in May 2018 and updated in October 2020.

Databases used were Scopus, PubMed, and ISI Web of Science, supplemented with a free search using Google Scholar. The database search query was composed of three search concepts: ‘population’ (adults 50 years+), AND ‘instrument’ (tools/questionnaires/measurement) AND ‘context’ (developing countries), AND ‘outcome’ (quality of life). Per search concept, free text words were used, all separated by the Boolean operator “OR”. The free words used for the population included: “frail elderly”, “elder*”, “senior*”, “older person*”, “old people”, “aged”, “aged, 50 and over”, “septuagenarian*”, “nonagenarian*”, “octogenarian*” and “centenarian*”. The free words used for the instrument included “instrument*”, “tools*”, “questionnaires*” and “measurement tool*”. The free words used for the context included: “developing countr*”, “third-world nation*”, “under developed countr*”, “less developed nation*”, “developing nation*”, “Africa” and “subsahar*” The free words used for the outcome included: “Activities of Daily Living”, “ADL”, “quality of life”, “QoL”, “Health-Related Quality Of Life”. Hand searching and screening of references was done after inclusion of a full text.

### Study selection

Studies were included in the review if they met the following criteria:

- Research that used generic QoL instruments among non-disease specific populations;
- Studies that use a multi-domain instrument to assess QoL, including at least the physical, psychological and social domain;
- Research focused on populations of adults which are on average 50 years and above;
- Publications written in English;
- Articles published since 2008 (in order to reflect recent research developments);
- Research conducted in African countries.

Studies using secondary data, case studies or conference abstracts were excluded, as well as studies of which the full articles were not attainable, also after contacting authors.

Even though in this publication we are interested in finding QoL instruments which could be used in slum settings, research done in the slum setting was not used as an inclusion criteria. The reason for this was that imposing this restriction led to limited results.

Authors PYAA and IHJE developed the literature search with the assistance of a librarian. After performing the search in the databases, all titles and abstracts were reviewed based on the inclusion and exclusion criteria. Articles which met the inclusion criteria were obtained in full-text and reviewed for final eligibility by author PYAA. Author IHJE checked if she agreed whether or not the final selection of articles met the inclusion criteria. The reference lists of the included articles were also hand searched to see if any studies were missed in the initial search.

### Data extraction

A data extraction sheet was developed by the authors including the following categories: authors, year of publication, title of article, country, study design, population (number of participants, minimum age), setting and name of instrument used to assess QoL (Table 1). First author PYAA conducted the data extraction.

**Table 1:** Characteristics of included studies

	Authors, year	Title	Country	Study design	Population, minimum age	Setting	QoL instrument
1	Xavier Gómez-Olivé et al., 2010 [14]	Assessing health and well-being among older people in rural South Africa.	South- Africa	Secondary data analysis of WHO- SAGE study	• *n* = 4085 • Age = > 50 years	Community	EUROHIS-QOL-8
2	Wilunda, et al., 2015 [15]	Health and ageing in Nairobi’s informal settlements- evidence from the INDEPTH: a cross sectional study	Kenya	Secondary data analysis of WHO- SAGE and INDEPTH study	• *n* = 1878 • Age > 50 years	Community /Slum	EUROHIS-QOL-8
3	Kyobutungi, et al., 2010 [16]	The health and well-being of older people in Nairobi’s slums	Kenya	Secondary data analysis on NUHDSS database	• *n* = 2072 • Age > 50 years	Slum	EUROHIS-QOL-8
4	Xavier Gómez-Olivé et al., 2014 [17]	Social conditions and disability related to the mortality of older people in rural South Africa.	South- Africa	Secondary data analysis of WHO- SAGE study	• *n* = 4047 • 75.2% female • Age > 50 years	Community	EUROHIS-QOL-8
5	Xavier Gómez-Olivé et al., 2013 [18]	Self-reported health and health care use in an ageing population in the Agincourt sub-district of rural South Africa.	South- Africa	Secondary data analysis of WHO- SAGE study	• *n* = 425 • 66.8% female • Age > 50 years	Community	EUROHIS-QOL-8
6	Mwanyangala, et al., 2010 [19]	Health status and quality of life among older adults in rural Tanzania	Tanzania	Secondary data analysis of SAGE study	• *n* = 5131 • Age > 50 years	Community	EUROHIS-QOL-8
7	Ralston et al., 2019 [20].	Policy shift: South Africa’s Old Age Pensions’ Influence on Perceived Quality of Life	South- Africa	Secondary data analysis of SAGE study	• *n* = 9341 • Age > 50 year	Community	EUROHIS-QOL-8
8	Macia et al., 2015 [21]	Exploring Life Satisfaction Among Older Adults in Dakar.	Senegal	Cross sectional study	• *n* = 500 • Age > 50 years	Community	SWLS
9	Gureje et al., 2014 [22]	Profile and determinants of successful aging in the Ibadan Study of Ageing.	Nigeria	Secondary data analysis of longitudinal study	• *n* = 930 • 38.9% Female • Age > 65 years	Community	SWLS
10	Gutiérrez et al., 2013 [23]	Predicting life satisfaction of the Angolan elderly: a structural model.	Angola	Cross sectional study	• *n* = 1003 • 65.4% females • Age > 60 years	Long-term care facilities	SWLS
11	Van Biljon et al., 2015 [24]	A partial validation of the WHOQOL-OLD in a sample of older people in South Africa	South- Africa	Cross sectional study	• *n* = 176 • 71.6% female • Age > 60 years	Long Term Facilities	WHOQOL-OLD
12	Akosile et al., 2018 [25]	Depression, functional disability and quality of life among Nigerian older adults: Prevalences and relationships.	Nigeria	Cross sectional study	• *n* = 206 • 56.3% female • Age > 65 years	Community	WHOQOL-OLD
13	Mugomeri et al., 2017 [26]	Quality of Life of the Elderly Receiving Old Age Pension in Lesotho.	Lesotho	Cross sectional study	• *n* = 385 • Age > 70 years	Community	WHOQOL-BREF
14	Gureje et.al 2010 [27]	Determinants of quality of life of elderly Nigerians: results from the Ibadan Study of Ageing	Nigeria	Longitudinal study	• *n* = 2175 • Age > 65 years	Community	WHOQOL-BREF
15	Akosile et al., 2014 [28]	Fear of Falling and Quality of Life of Apparently-Healthy Elderly Individuals from a Nigerian Population	Nigeria	Cross sectional study	• *n* = 261 • 49.8% female • Age > 65 years	Community	SF-36
16	Ogunyemi et al., 2018 [29]	Health-Related Quality of Life of the Elderly in Institutional Care and Non-Institutional Care in Southwestern Nigeria: A Comparative Study.	Nigeria	Cross sectional study	• *n* = 360 • Age > 60 years	Community	SF-36
17	Younsi, 2015 [30]	Health-Related Quality-of-Life Measures: Evidence from Tunisian Population Using the SF-12 Health Survey	Tunisia	Cross sectional study	• *n* = 3864 • 51.9% Female • Age 18–85 years (50–59: *n* = 711; 60–74: *n* = 580; 75–85: 224)	Community	SF-12
18	Ramocha et al., 2017 [12]	Quality of life and physical activity among older adults living in institutions compared to the community	South- Africa	Cross sectional study	• *N* = 80 • 42.5% • Age > 60	Community and nursing home	RAND-36

*EUROHIS-QOL* 8-item WHOQOL questionnaire; *WHOQOL* World Health Organization Quality of Life Scale; *SWLS* Satisfaction with Life Scale; *WHOQOL-OLD* World Health Organization Quality of Life Scale- OLD version; *WHOQOL-BREF* World Health Organization Quality of Life Scale- brief version; *SF-36* Short-Form Health Survey-36 item; *SF-12* Short-Form Health Survey 12-item; *SAGE study* Study on global AGEing and adult health; *INDEPTH* International Network for the continuous Demographic Evaluation of Populations and Their Health in developing countries; *NUHDDS* longitudinal Nairobi Urban Health and Demographic Surveillance System;

Next, the QoL instruments found were further assessed on number of items, domains included in the instrument, reliability, validity, language of the instrument, mode of administration (interviewer administered or self-administered) and answer categories (open answers / Likert scales / VAS scales or pictorial scales). All types of reliability reported in the included articles were taken into account and described in the data extraction sheet. For internal reliability, a Cronbach’s alpha score of > 0.70 was considered a good score. Furthermore, all types of validity reported in the included articles were taken into account and described in the data extraction sheet. When the included articles did not report on reliability or validity of the instrument used in their country and/or study population, this was described as ‘ND’ (Not Described).

## Results

**Fig. 1:**
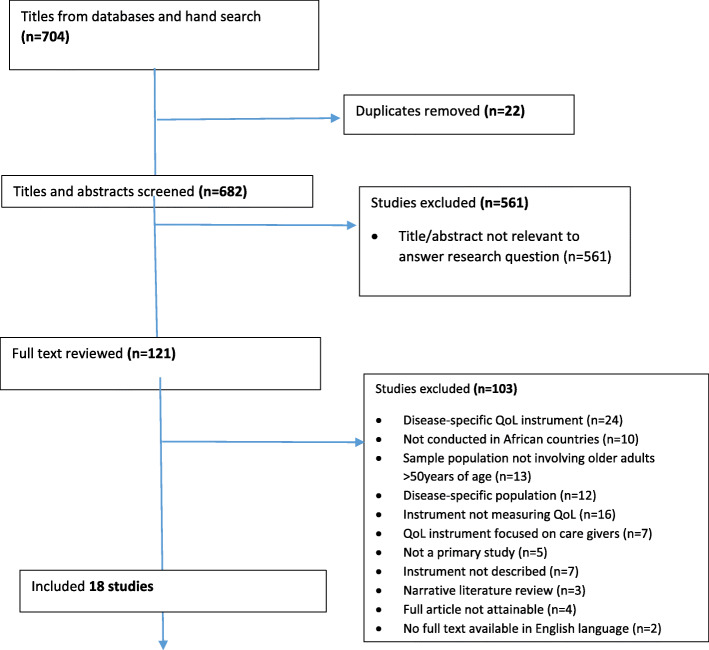
Schematic flow of search results

Figure 1 shows a summary of the screening and inclusion process. Databases and hand searches revealed an initial 704 records. After duplicate records (*n* = 22) were removed, 682 records remained. Screening of titles and abstracts of these records resulted in the exclusion of 561 records that did not meet the inclusion criteria. In total, 121 articles were retrieved for full-text assessment. After screening these full-text articles, 103 articles were excluded, as they did not meet all inclusion criteria. All reasons for exclusion can be found in Fig. 1. The final number of articles included in this review was 18. In total, these studies assessed QoL using seven unique instruments. The main characteristics of the 18 studies included are shown in Table 1.

### Research design

Out of the 18 studies included, nine studies (50%) used a cross-sectional design [12, 21, 23, 24, 26, 28–30] while eight studies (44%) performed a secondary data analysis of collected cross-sectional or longitudinal data [14–20, 22] and one study (6%) used a longitudinal design [27].

### Participants and settings

The number of participants included in the studies varied from 80 [12] to 9341 subjects [20], with a total of 36,919. All participants were above the age of 50. Most studies were conducted in South Africa (*n* = 6) [12, 14, 17, 18, 20, 24] and Nigeria (*n* = 5) [22, 25, 27–29], followed by Kenya (*n* = 2) [15, 16]. From the countries Tanzania [19], Senegal [21], Angola [23], Lesotho [26] and Tunisia [30], in each case, one study was included in this review. The community setting accounted for 14 of the included studies, two studies were performed in nursing homes or long-term care facilities [23, 24] and one study had a mix of nursing home residents and community dwelling older adults [12]. Two of the studies [15, 16] specifically studied slum settings.

### Instruments assessing QoL

In this review, 7 different instruments were found in 18 different studies (Table 1). The most often used instruments was the “EUROHIS-QOL” (*n* = 7). This is an eight-item instrument derived from the WHO-QOL-BREF. The “Satisfaction with Life Scale” (SWLS) was used in 3 studies. The World Health Organization Quality of Life Scale-brief version” (WHOQOL-BREF), the “World Health Organization Quality of Life Scale-old version” (WHOQOL-OLD) and the Short-Form Health Survey-36 (SF-36) were all used twice. One study made use of the “Short-Form Health Survey-12 (SF-12; *n* = 1) and the other used the RAND-36 [12], which is equal to the SF-36 but using a different scoring system. The two studies that included slum settings both used the EUROHIS-QOL to assess QoL of participants [15, 16]. The instruments “EUROHIS-QOL”, “WHOQOL-BREF” and “WHOQOL-OLD” are all derivatives of the WHOQOL-100″,

### Instrument domains

As shown in Table 2, the number of items per instrument varied between 5 and 36. Domains included in all instruments were the physical, psychological and social domain. In the EUROHIS-QOL-8, WHOQOL-OLD, WHO-QOL-BREF, SF-36, SF-12 and RAND-36, these domains are directly captured by asking specific questions such as ‘How satisfied are you with your ability to perform your daily living activities?’ (Physical domain, EUROHIS-QOL-8) or ‘to what extent do you experience limitations in usual role activities because of emotional problems?’ (Psychological domain, SF-36), ‘how satisfied are you with your personal relationships?’ (Social domain, WHOQOL-BREF) or “how satisfied are you with the conditions of your living place?” (Environmental domain, WHOQOL-BREF). The SLWS asks questions regarding general life satisfaction, using 5 statements that have to be assessed on a scale of 1–7. An example statement is ‘In most ways, my life is close to my ideal’.

**Table 2:** Characteristics of QoL instruments

QOL instrument	No. of studies	No. items	Domains	Reliability	Validity	Languages	Mode of administration	Answer categories
EUROHIS-QOL-8 [14–20]	7	8	• physical; • psychological; • social; • environmental	ND for study cohorts under consideration	ND for study cohorts under consideration	• Shangaan [14, 17] • Xitsonga [18] • Local language Nairobi (Kenya) [15, 16] • Local language in Agincourt (South-Africa) [20] • Kiswahili [19]	• IA [14–20]	5- point Likert scale
SLWS [21–23]	3	5	Satisfaction with life as a whole	Internal reliability: • α = 0.82 [21] • α = 0.81 [22] • α = 0.92 [23]	• Good content validity based on expert meeting [21] • ND [22, 23]	• Wolof [21] • Yoruba [22] • Portugese [23]	• IA [21] • ND [22] • IA/SA [23]	7-point Likert scale
WHOQOL-OLD [24, 25]	2	24	• sensory abilities • autonomy • past, present, and future activities • social participation • death and dying • intimacy	• Per domain α ranging from 0.72 to 0.84 [24] • ND for this study cohort [25]	• Good factor structure [24] • ND for this study cohort [25]	• Afrikaans [24] • Unknown [25]	• SA/IA [24] • IA [25]	5-point Likert scale
WHOQOL-BREF [26, 27]	2	26	• physical; • psychological; • social; • environmental	• Internal consistency each domain α > 0.67 [26] • Cronbach α > 0.86 [27]	ND for this study cohort [26, 27]	• Sesotho [26] • Yoruba [27]	• IA [26] • SA/IA [27]	5- point Likert scale
SF-36 [28, 29]	2	36	• physical health o physical functioning o physical role limitation o bodily pain o general health • mental health: o vitality o social functioning, o role limitation due to emotional problems o mental health	ND for this cohort [28, 29]	ND for this cohort [28, 29]	• Unknown [28]	• IA [28]	Mix of 5 / 3 – point Likert scale and yes/no answers
SF-12 [30]	1	12	• physical health o physical functioning o physical role limitation o bodily pain o general health • mental health: o vitality o social functioning, o role limitation due to emotional problems	Internal reliability: • Physical health α = 0.76 • mental health α = 0.74 [30]	• Construct validity good (differences between subgroups) • convergent validity good: (r > 0.40) [30]	Tunisian [30]	IA [30]	Mix of 5 / 3 – point Likert scale and yes/no answers
RAND-36 [12]	1	36	• physical functioning • bodily pain • limitation because of physical health problems • role limitation because of personal or emotional problems • emotional well-being • social functioning, • energy or fatigue • general health perception.	Not assessed (only refer to reliability of SF-36 tested in Ghanese setting where α = 0.82) [12]	Description of adequate face and content validity [12]	Setswana and isiZulu [12]	IA [12]	Mix of 5 / 3 – point Likert scale and yes/no answers

*ND* Not Described; *IA* Interviewer Administered; *SA* Self-Administered; *EUROHIS-QOL-8 WHOQOL* World Health Organization Quality of Life Scale; *SWLS* Satisfaction with Life Scale; *WHOQOL-OLD* World Health Organization Quality of Life Scale- OLD version; *WHOQOL-BREF* World Health Organization Quality of Life Scale- brief version; *SF-36* Short-Form Health Survey-36 item; *SF-12* Short-Form Health Survey 12-item

Besides the domains mentioned above, the WHOQOL-OLD includes domains of importance to older adults, such as ‘sensory abilities’ or ‘death and dying’. The SF-36 and SF-12 are health related quality of life instruments (HRQOL), asking to what extent one’s health interferes with e.g. physical function, mental health or social functioning. The environmental domain was only captured in the EUROHIS-QOL-8 and the WHOQOL-BREF.

### Reliability and validity of instruments

Only 7 studies provided figures on reliability. All studies using the SLWS showed good internal reliability (Cronbach’s Alpha ≥0.81) [21–23], one study showed good internal reliability of the WHOQOL-OLD (Cronbach’s Alpha ≥0.72) [24] and two studies showed moderate to good internal consistency on the WHOQOL-BREF (Cronbach’s Alpha ≥0.67 [26] and ≥ 0.85 [27]). One study described good internal reliability of the SF-12 (Cronbach’s Alpha per domain ≥0.74) [30]. There were no descriptions on reliability in the studies using the EUROHIS-QOL-8 and the SF-36. The study using the RAND-36 did describe good validity of the comparable SF-36, but this was tested in another African country in a different target group [12]. Scores on other forms of reliability were not provided in any of the studies.

Only four studies described results on validation. Macia et al., using the SLWS, described good content validity based on an expert meeting [21]. Van Biljon et al., described a good factor structure when using the WHOQOL-OLD [24] and Younsi et al., looking at the validity of the SF-12 in a Tunisian population, described a good construct and convergent validity [30]. Lastly, Ramocha et al. described adequate face and content validity but did not give more information on how this was assessed [12]. All other studies did not describe anything about validation of the instrument in their specific cohorts or countries.

### Suitability of using instrument among illiterate population

Seventeen studies described that the instrument was interviewer administered, or could be interviewer administered. One study did not provide information on the mode of administration [22]. All instruments used Likert scale or yes/no answer categories and no instruments made use of VAS scales or pictorial scales.

## Discussion

In this review, 7 different instruments in 18 studies were found to measure QoL among older adults in African countries: the EUROHIS-QOL-8 (*n* = 7), the SWLS (*n* = 3), the WHOQOL-OLD (*n* = 2), the WHOQOL-BREF (n = 2), the SF-36 (n = 2), the SF-12 (*n* = 1) and the RAND-36 (n = 1). After careful reflection on their respective psychometric properties, it appeared that the SLWS had good internal validity. The other instruments, had little or no information was available about the psychometric properties of the instruments when using them in their specific countries or populations. As not all aspects of life are equally important for all age groups, cultures and settings, it cannot be automatically assumed that all instruments are applicable for its intended purpose [31]. All studies using the EUROHIS-QOL-8 to assess QoL used data from the WHO Study on Global AGEing and Adult Health (SAGE study) refer to Kowal et al. [32] and Schmidt et al. [33] for information on the psychometric properties. Although the conclusion of Schmidt et al. is that the EUROHIS-QOL-8 showed good cross-cultural field study performance and a satisfactory convergent and discriminant validity, this was only assessed in European countries [33]. Furthermore, despite the fact that the EUROHIS-8 instrument is a derivate of the cross-culture validated WHOQOL-BREF, it is unknown if this instrument also shows good psychometric properties when using it in developing countries and more specifically in slum settings. Lastly, the seven studies using the EUROHIS-QOL-8 only reported on outcome and not on process-related measures such as experiences during data collection or feasibility of using the scale in the specific (slum) setting.

As a large proportion of older adults in African countries live in slums and are illiterate, this study also reviewed if the instruments were suitable to use among an illiterate population. Even though there is little information available on time investment when administering the instruments, the number of items per instrument range from 5 to 36, which seems like a relatively permissible time investment. When looking at mode of administration, one study did not describe how the instrument was administered [22] but all other studies described the possibility of interviewer-administration. However, there appeared to be no instrument which made use of VAS scales or pictorial scales, which could have made application in an illiterate population easier. *Something else that would make the use of instruments more feasible is the possibility of amendment of questions (to make it understandable to the illiterate population) in the instruments* to improve suitability of the instrument towards the population or setting (e.g. climbing stairs in a slum setting is not common or performing heavy exercise is less common among an older population). However, doing this will have consequences for performance characteristics of the instrument and should therefore be performed with caution. The original meaning should not change so as to measure the intended items in the domains.

When looking at the domains included in the different QoL instruments found in our review, the physical, psychological and social domain appeared to be (indirectly) included in almost all instruments. The environmental domain, however, was only included in the EUROHIS-QOL-8 and the WHOQOL-BREF. However, the environmental domain is likely to be of importance in African countries and more specifically in slum settings due to factors mentioned in the introduction paragraph of this article, such as poor housing, lack of sanitation and environmental hazards such as improper disposal of waste. A study performed among adolescents in Bangladesh also showed worse scores on physical environment and QoL among slum dwellers compared to non-slum dwellers [34]. Also, a study by Nilsson et al. [35], a literature search was performed to identify appropriate instruments to assess health related quality of life (HRQoL) among older people in rural Bangladesh. In addition, in-depth interviews with these older adults were performed to retrieve information on QoL domains deemed important by the older adult population. This study concluded that the instruments which were found to assess HRQoL mainly looked at physical, psychological and social domains, while older adults stated that spiritual, economic and environmental domains are equally important but not present in these instruments [50]. Also, some studies argue that in low- and middle-income countries, quality of life is more described in family and group terms, including values such as interdependence and role fulfilment instead of e.g. autonomy. These concepts might currently be underrepresented in QoL instruments [36].

From these findings, it can be concluded that the majority of the instruments found to assess QoL in African countries can be interviewer administered and are relatively short, providing a good starting point for use in an illiterate population. However, the instruments lack basic information on reliability and/or validity and more information is needed to know if the domains used in the different instruments actually reflect quality of life of older adults living in African slums.

### Strengths and limitations

To the authors’ best knowledge, this is the first scoping review which identified and critically reflected on instruments used to assess QoL of older adults living in African countries. Therefore, this review provides valuable new insights into instruments used to measure quality of life in African countries. Another strength of this study is the fact that framework of Levac and colleagues on advancing the method of scoping reviews were followed, resulting in an excellent methodological foundation of this scoping review [13].

However, some limitations should also be mentioned. First, studies reporting on quality of life among disease-specific populations and studies describing the development of QoL instruments were excluded from this review. There is a possibility that these studies used generic quality of life instruments that could have contributed to our results. However, as we were looking for instrument applicable in the general older population and to not development but mainly feasibility of applying the instrument in the specific settings, it was decided to exclude those publications. Second, only two articles were found describing the use of a QoL instrument in the slum setting and these studies did not provide any data on psychometric properties and feasibility of using the scale in the slum setting. As a consequence, very little conclusions can be drawn on the use of QoL instrument in older adults living in slums. Lastly, all articles were excluded that were written in another language than English. As the focus of our study was quality of life in African countries, this might have led to missing publications written in another language.

For this reason, excluding non-English articles could also be a limitation.

### Recommendations for future research

As there is little information available on the psychometric properties of the instruments used to assess QoL of older adults in African countries, further research should focus on validation and reliability of the instruments used among this specific population. Furthermore, there is a need for more in-depth research on the content and domains of instruments to assess QoL among older adults in African countries, and more specifically, in slum settings. Only two studies performed QoL measurements in slum settings but did not describe anything about their experiences of using the scale. Therefore, it is also recommended that in-depth interviews are carried out among aged slum dwellers to see if the content of currently used QoL instruments matches the concept of QoL experienced by them. A possibility that could also be explored is the weighing of specific domains based on importance valued by respondents [36]. Finally, further research should not only look at content of the instruments but also at its feasibility to use in an illiterate population. Aspects such as how easily an illiterate population can understand (e.g. short and clear questions, pictorial scales or only performing in-depth interviews) and time investment are important aspects to explore.

## Conclusion

The aim of this scoping review was to synthesize the current body of knowledge on the instruments used to assess quality of life of older adults in African countries. The following instruments were found: the EUROHIS-QOL-8, the SLWS, the WHOQOL-OLD, the WHOQOL-BREF, the SF-36 and the SF-12. It appeared that little information is available on both psychometric properties and feasibility of using these instruments among older adults in African countries. Also, it is unknown if the domains used in the instruments actually reflect quality of life in this specific population. This highlights the need for further research on adjustment and validation of currently existing instruments and/or for the development and validation of a new instrument, which can be used in illiterate, older populations living in African slums.

## Data Availability

All data generated or analyzed during this study are included in this published article [and its supplementary information files].

## Abbreviations

ND: Not Described
IA: Interviewer Administered
SA: Self-Administered
EUROHIS-QOL: 8-item WHOQOL questionnaire
SWLS: Satisfaction with Life Scale
WHOQOL-OLD: World Health Organization Quality of Life Scale- OLD version
WHOQOL-BREF: World Health Organization Quality of Life Scale- brief version
SF-36: Short-Form Health Survey-36 item
SF-12: Short-Form Health Survey 12-item
WHO-SAGE: World Health Organization - Study on Global Ageing and Adult Health
INDEPTH: International Network for the continuous Demographic Evaluation of Populations and Their Health in developing countries
NUHDDS: longitudinal Nairobi Urban Health and Demographic Surveillance System
